# Development and validation of a prognostic 9-gene signature for colorectal cancer

**DOI:** 10.3389/fonc.2022.1009698

**Published:** 2022-11-17

**Authors:** Junpeng Cui, Fangyu Guo, Yifan Yu, Zihuan Ma, Yuting Hong, Junyan Su, Yang Ge

**Affiliations:** ^1^ Department of General Surgery, Shengjing Hospital of China Medical University, Shenyang, Liaoning, China; ^2^ Department of Endocrinology, Shengjing Hospital of China Medical University, Shenyang, Liaoning, China; ^3^ Department of Scientific Research Projects, ChosenMed Technology Co. Ltd, Beijing, China

**Keywords:** colorectal cancer, risk score, prognosis, RNA sequencing, signature

## Abstract

**Introduction:**

Colorectal cancer (CRC) is one of the most prevalent cancers globally with a high mortality rate. Predicting prognosis using disease progression and cancer pathologic stage is insufficient, and a prognostic factor that can accurately evaluate patient prognosis needs to be developed. In this study, we aimed to infer a prognostic gene signature to identify a functional signature associated with the prognosis of CRC patients.

**Methods:**

First, we used univariate Cox regression, least absolute shrinkage and selection operator (lasso) regression, and multivariate Cox regression analyses to screen genes significantly associated with CRC patient prognosis, from colorectal cancer RNA sequencing data in The Cancer Genome Atlas (TCGA) database. We then calculated the risk score (RS) for each patient based on the expression of the nine candidate genes and developed a prognostic signature.

**Results:**

Based on the optimal cut-off on the receiver operating characteristic (ROC) curve, patients were separated into high- and low-risk groups, and the difference in overall survival between the two groups was examined. Patients in the low-risk group had a better overall survival rate than those in the high-risk group. The results were validated using the GSE72970, GSE39582, and GSE17536 Gene Expression Omnibus (GEO) datasets, and the same conclusions were reached. ROC curve test of the RS signature also indicated that it had excellent accuracy. The RS signature was then compared with traditional clinical factors as a prognostic indicator, and we discovered that the RS signature had superior predictive ability.

**Conclusion:**

The RS signature developed in this study has excellent predictive power for the prognosis of patients with CRC and broad applicability as a prognostic indicator for patients.

## Introduction

Colorectal cancer (CRC) is one of the most prevalent cancers globally, with a high mortality rate. CRC accounts for up to 10% of all new cancers and cancer-related deaths worldwide (1). The prognosis of CRC patients is currently determined by disease progression and tumor–node–metastasis (TNM) staging upon diagnosis (2). However, making predictions based solely on conventional prognostic factors is insufficient to fulfill the clinical demands (3).

Dysregulation of gene expression is a typical feature of cancer (4). Furthermore, RNAs, including protein-coding RNAs (mRNAs), long non-coding RNAs (lncRNAs), and microRNAs (miRNAs), play a crucial role in gene expression. This suggests that RNAs that regulate certain key cancer genes may play a role in cancer development (5). Several studies have demonstrated a relationship between aberrant RNA expression and prognosis of cancer patients. For example, high expression of gene amplified in esophageal cancer 1 (GAEC1) mRNA affects the clinical characteristics of CRC patients (6). The lncRNA FAM83H-AS1 has been associated with the progression of several cancers and can be utilized as a biomarker for cancer diagnosis and prognosis (7). Multiple genes are related to the prognosis of various types of cancer, and the identification of these prognosis-related genes would provide a relatively accurate prediction of the prognostic outcome of cancer patients (8). Previous studies have shown that risk score (RS) signatures with multiple prognosis-related genes effectively predict patient prognosis in cancers such as liver and lung cancer (9–11). Although previous studies have developed RS signatures for the prognosis of CRC patients, these studies have mainly focused on one type of RNA (12–14). Therefore, no restrictions were placed on the type of RNA used in this study to ensure the accuracy of prognosis prediction by the RS signature.

In this study, nine differentially expressed genes (DEGs) associated with the prognosis of CRC patients were analyzed, with data from The Cancer Genome Atlas (TCGA) database and used to develop a risk score for each patient and construct a RS signature. The results demonstrated that the 9-DEGs RS signature developed in this study could effectively predict the prognosis of CRC patients with greater predictive power than traditional clinical variables when validated using the Gene Expression Omnibus (GEO) database.

## Materials and methods

### Data source and clinical information download

RNA sequencing (RNA-Seq) expression data of 414 colorectal cancer samples were downloaded from TCGA (https://gdc-portal.nci.nih.gov/) database, which included 375 tumor samples and 39 normal tissue samples. Subsequently, the clinical information corresponding to the 414 samples was obtained. From this dataset, TCGA validation dataset of tumor samples (n = 89) was chosen at random, and the remaining tumor samples (n = 286) and 39 normal tissue samples were used as the training dataset in subsequent analysis. To verify the randomness of the selection process, the Wilcoxon rank-sum test and chi-square test were used to determine whether the distribution of the clinical indicators, including age, sex, tumor (T), node (N), metastasis (M), and TNM stage, differed between the TCGA validation and training datasets.

In addition, GSE72970, GSE39582, and GSE17536 RNA-Seq expression datasets (n = 124, n = 579, and n = 117, respectively) and the corresponding clinical information from the GEO (http://www.ncbi.nlm.nih.gov/geo/) database were downloaded and considered as three independent validation datasets. It is worth noting that 70% (87/124) of the patients in the GSE72970 dataset were stage III or IV cancer patients, as opposed to TCGA dataset.

### Identification and screening of differentially expressed genes (DEGs)

Differentially expressed genes between tumor and normal tissue samples from the training dataset were identified using DESeq2 package (version 1.34.0) (15). The p-values were adjusted using FDR method in our study. The genes that met the filtering criterion of |log 2FC| >1 and *adjusted p*< 0.05 were classified as DEGs. The Pheatmap (16) package (version 1.0.12) and the ggplot2 (17) package (version 3.3.5) were used to create a heatmap and volcano map, respectively, to show the identified DEGs. All data processing and analyses were performed using R version 4.1.2.

### Functional and pathway enrichment analysis

The gene ontology (GO) and Kyoto Encyclopedia of Genes and Genomes (KEGG) analysis were performed using the clusterProfiler (18) package (version 4.2.2) and the org.Hs.eg.db (19) package (version 3.14.0). GO and KEGG terms and pathways with *adjusted p*< 0.05 were considered significant. Gene Set Enrichment Analysis (GSEA) was performed using the same packages.

### Prognostic RS signature construction

First, the survival (20) package (version 2.41.1) was used to perform a univariate Cox regression analysis on the screened DEGs; genes with *adjusted p*< 0.05 were considered highly associated with prognostic outcomes in CRC patients and were retained. Subsequently, least absolute shrinkage and selection operator (Lasso) regression analysis was performed to further screen important genes using the glmnet (21) package (version 4.1.3). Then, a multivariate Cox regression analysis was performed to evaluate the independence of the screened genes, and the rms (22) package (version 6.2.0) was used to detect collinear genes and filter them out (variance inflation factor (VIF) > 2) (23). The remaining genes were considered candidate genes for the prognostic RS signature.

The risk score for each patient was calculated according to the following formula:


$$\text{Risk score }(\text{RS})=\sum_{i=1}^{N}(\text{Coef}(\text{i})*\text{Exp}(\text{i}))$$

Where N represents the number of candidate genes, Coef represents the coefficient value of candidate genes in the multivariate Cox regression analysis, and Exp represents the expression level of candidate genes. After the risk score for each patient was calculated, the survminer (24) package (version 0.4.9) was used to determine the optimal cut-off value based on the risk score and patient prognosis. The patients in the training dataset were separated into two categories based on this cut-off value: high risk and low risk. TCGA validation dataset and three GEO validation datasets also used the above method to calculate the risk scores and were divided into high- and low-risk groups based on the cut-off value.

### Protein expression analysis of candidate genes

The Human Protein Atlas (HPA) is a database of immunohistochemistry (IHC)-based protein expression profiles of different cancers, normal tissues and cell lines. To analyze the protein expression of candidate genes, we obtained IHC staining images of candidate genes in tumor and normal tissues from the HPA database (https://www.proteinatlas.org/).

### Functional and pathway enrichment analysis for RS-related DEGs

Next, we performed functional and pathway enrichment analysis for RS-related DEGs using the aforementioned method, and GO and KEGG terms and pathways with *adjusted p*< 0.05 were considered significant.

### Validation of the RS signature

Survival analysis was performed on the high- and low-risk groups of all datasets using the survival (25) (version 3.3.1) and survminer packages, with death as the event; the time interval from the detection of the tumor to the occurrence of the event or last follow-up date was considered as survival time. The difference in survival between the high- and low-risk groups was demonstrated using a Kaplan-Meier (KM) survival curve. Furthermore, we used the receiver operating characteristic (ROC) curve to assess the overall predictive accuracy of the RS signature in the training dataset and the area under the ROC curve (AUC) was considered an indicator of the predictive performance of the RS signature for patient prognostic outcomes, using the survivalROC (26) package (version 1.0.3). The disease-free survival (DFS) of patients in the training dataset was also analyzed using the same method to examine the difference in prognostic outcomes between the high- and low-risk groups. To explore the predictive ability of RS signature in patients with colon and rectal cancer, we further selected patients with colon and rectal adenocarcinoma from the TCGA dataset and analyzed the performance of RS signature in predicting the prognosis of patients using KM and ROC curves. Subsequently, using univariate and multivariate Cox regression analyses with the training dataset, the risk score was identified as an influential factor in a patient’s prognosis compared to the patient’s age, sex, and tumor pathological stage. To better evaluate the value of RS in clinical decision making, decision curve analysis (DCA) was performed using ggDCA (27) package (version 1.2) and the area under decision curves (AUDC) was used to characterize the value of RS and other clinical indicators, including age, sex, T, N, M, and TNM stage.

To investigate the differences between the high- and low-risk groups at the gene expression level, we identified differentially expressed genes between those two groups using the abovementioned approach and plotted the results into volcano plot and heatmap. We then performed functional and pathway enrichment analyses of the RS-related DEGs. Subsequently, CIBERSORT (28) algorithm and LM22 immune cell reference gene expression matrix were used to investigate differences in immune infiltration between the high- and low-risk groups. Next, we examined 32 key immune checkpoints (**Supplementary Table 1**) in the training dataset to examine differences in the expression of immune checkpoints between the two groups, as well as immune escape.

### Drug sensitivity

The Genomics of Drug Sensitivity in Cancer (GDSC) database and the oncoPredict (29) package (version 0.2) were used to calculate the half maximal inhibitory concentration (IC50) to derive the difference in drug sensitivity between high and low risk groups of patients.

## Results

### DEGs in TCGA training dataset

**Figure 1:**
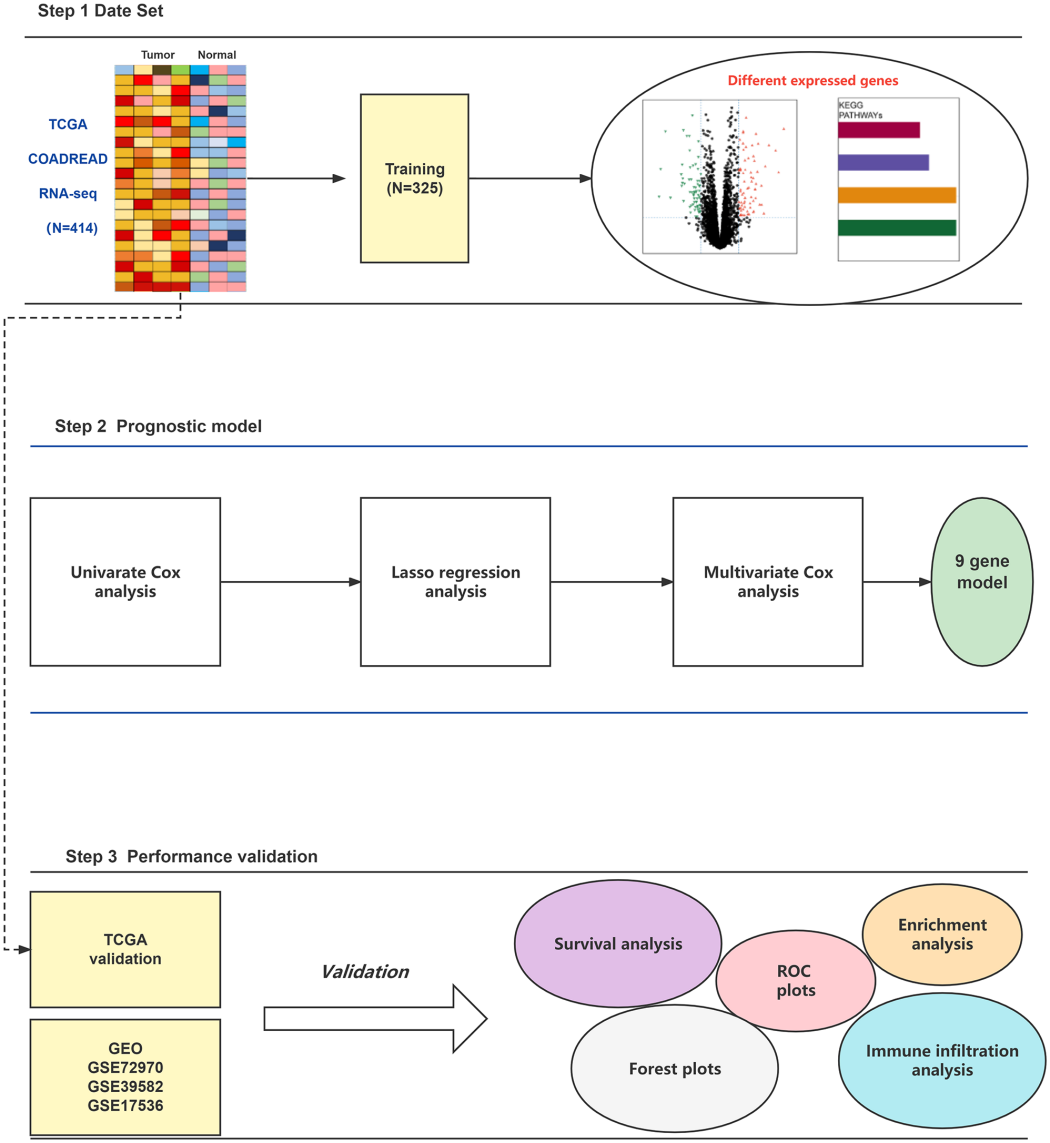
The workflow of this study.

**Figure 2:**
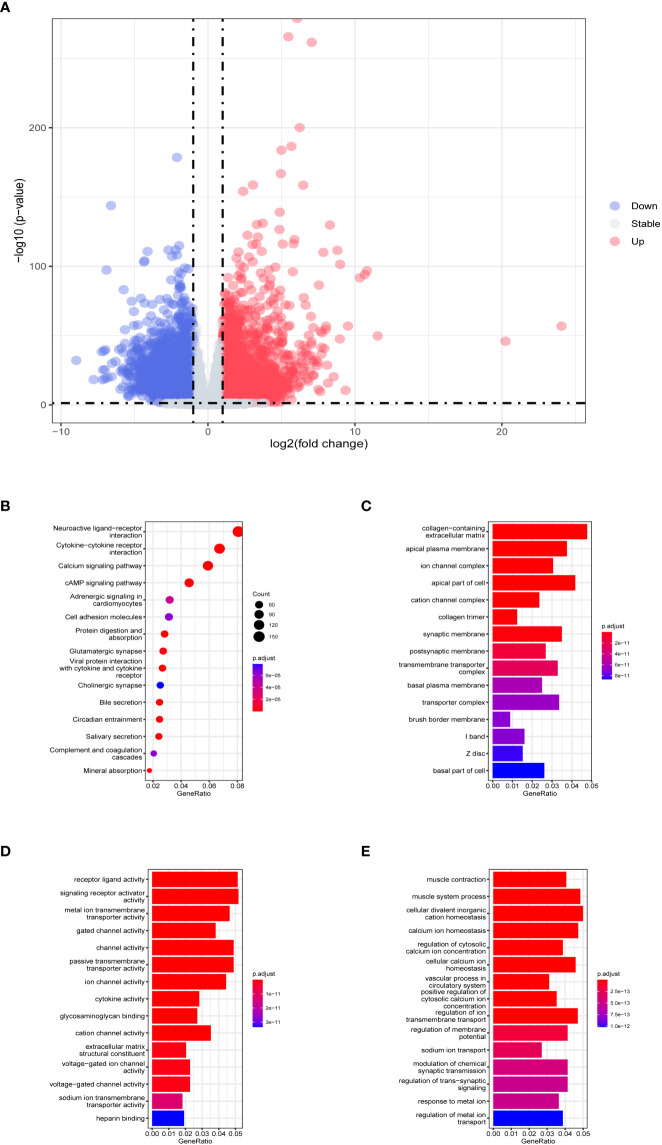
Evaluation of TCGA training dataset DEGs. **(A)** Volcano plot of DEGs in TCGA training dataset. The horizontal coordinate is log2 (fold change), with the distribution of genes with large differences at either end of the horizontal coordinate. The vertical coordinate is -log10 (*p*-value), with larger *p*-values indicating more significant differences. **(B)** Results of KEGG analysis of DEGs. The horizontal coordinate representing the ratio of a pathway’s genes to the total genes. The vertical coordinate is the name of the enriched pathways. The size of the circle represents the number of genes enriched by that pathway. The color represents the *p*-value, the redder the color the larger the *p*-value. Histograms of **(C)** cellular component, **(D)** molecular function, and **(E)** biological process in GO analysis. The horizontal coordinate is generated, representing the ratio of a term’s genes to the total genes. The vertical coordinate is the name of the enriched terms. The color represents the *p*-value, the redder the color the larger the *p*-value.

The flow chart of the overall analysis is shown in **Figure 1**. From the training dataset, 5286 DEGs were identified in 286 tumor samples and 39 normal tissue samples (detailed in **Supplementary Table 2**). The expression of DEGs is shown in a volcano plot (**Figure 2A**). The expression heatmap of the DEGs showed significant differences in gene expression levels between tumor and normal tissue samples (**Supplementary Figure 1**). The results of the functional and pathway enrichment analyses are shown in **Figures 2B–E**. In total, 74 significantly enriched KEGG pathways were detected, and 1838 GO terms (*adjusted p*< 0.05), including 151 cellular component (CC) terms, 220 molecular function (MF) terms, and 1467 biological process (BP) terms were identified as significantly enriched. Neuroactive ligand-receptor interaction was the most significant pathway in the KEGG analysis. Collagen-containing extracellular matrix, receptor-ligand activity, and muscle contraction were the most important phrases for cellular component, molecular function, and biological processes, respectively. GSEA resulted in 19 significant pathways, with 14 in the low expression genes (**Supplementary Figure 2A**) and 5 in the high expression group (**Supplementary Figure 2B**). The results of Wilcoxon rank-sum test and chi-square test (**Supplementary Table 3**) indicated that there was no statistical difference in the distribution of clinical indicators in the TCGA validation and training datasets, demonstrating the randomness of selecting tumor samples to form the TCGA validation dataset.

### Evaluation of the association between DEGs and overall survival (OS) of CRC patients

All the 5286 DEGs **(** **Supplementary Table 2**)were subjected to univariate Cox regression analysis, among which 608 DEGs passed the threshold (*adjusted p*< 0.05) and advanced to the next step of Lasso regression analysis **(** **Supplementary Figure 3**), where nine DEGs were selected for further screening using multivariate Cox regression analysis. The final 9-DEGs qualified by the collinearity test (**Supplementary Figure 4**) were selected and constructed as the RS signature. The results of univariate and multivariate Cox regression analyses for the 9-DEGs are displayed in **Table 1**.

**Table 1:** Univariate and Multivariate Cox analysis results.

Univariate Cox analysis						Multivariate Cox analysis				
gene	coef	exp (coef)	se (coef)	z	Pr (>|z|)	coef	exp (coef)	se (coef)	z	Pr (>|z|)
CPT2	-0.95363	0.38534	0.000031	0.000031	0.000033	-0.54387	0.580496	0.248541	-2.18826	0.028651
PPARGC1A	-0.23485	0.790691	0.000163	0.000163	0.000397	-0.13868	0.870508	0.07325	-1.89322	0.058329
LBX2	0.444483	1.559683	0.000325	0.000325	0.000525	0.175724	1.192109	0.146462	1.19979	0.230221
PANX2	0.321049	1.378573	0.000067	0.000067	0.000126	0.128094	1.13666	0.094177	1.360145	0.173784
LOC339674	0.413144	1.511562	0.000306	0.000306	0.000423	0.302522	1.353267	0.119869	2.523761	0.011611
GPR156	0.562897	1.755751	0.000104	0.000104	0.000273	0.11394	1.120685	0.155988	0.730443	0.46512
PPFIA4	0.364203	1.439367	0.000096	0.000096	0.000105	0.233869	1.26348	0.104617	2.23548	0.025386
LRP2	0.337768	1.401815	0.000121	0.000121	0.000579	0.172185	1.187898	0.088653	1.942244	0.052108
MBL1P	0.636906	1.890622	0.000011	0.000011	0.00002	0.42619	1.531412	0.153068	2.784326	0.005364

**Figure 3:**
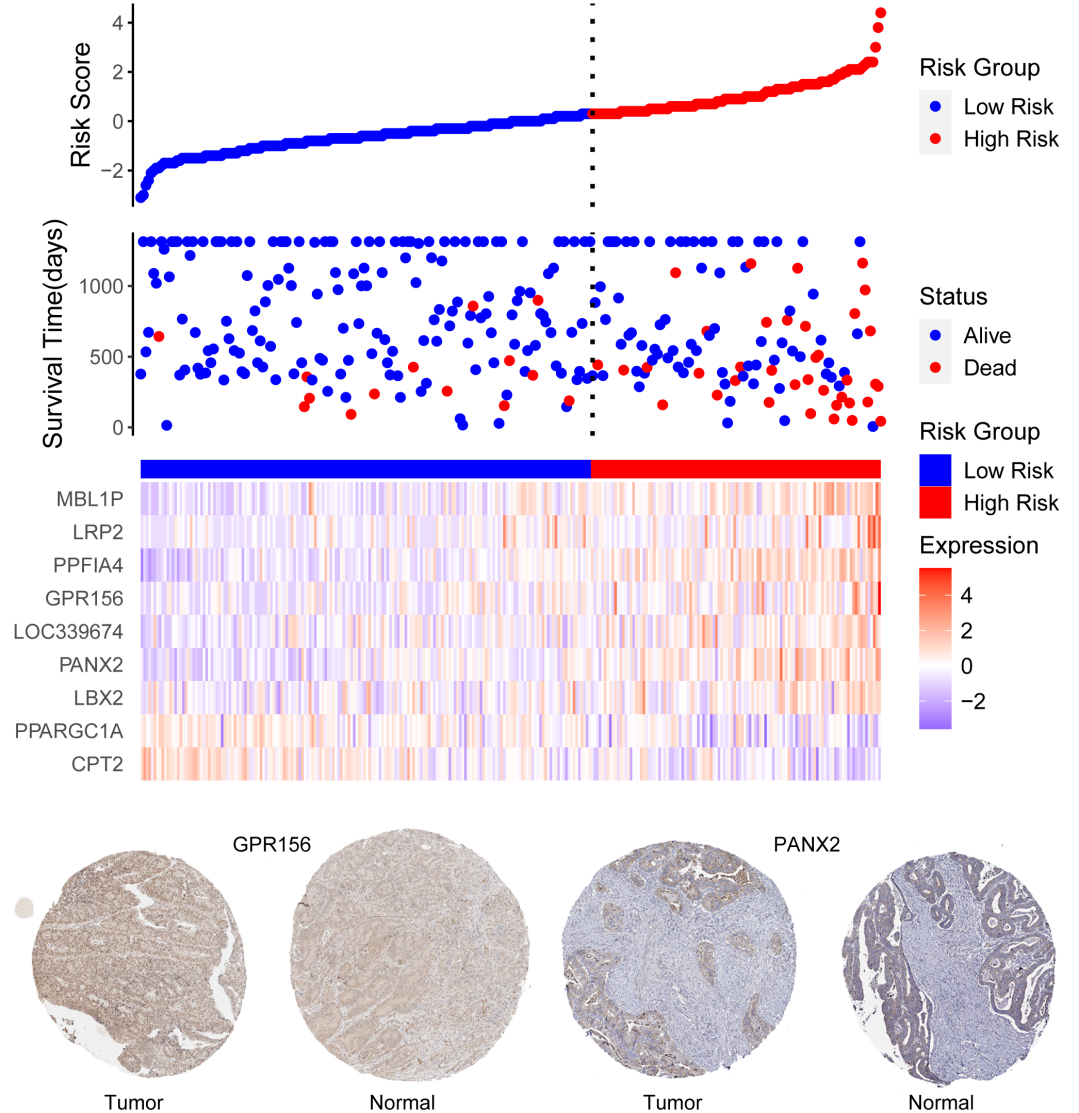
**(A)** Distribution of risk scores, **(B)** patient survival status, and **(C)** heat map expression differences of 9-DEGs in TCGA training dataset. The horizontal coordinates are patient IDs ranked by risk score from smallest to largest. The vertical coordinates are risk score, survival time (days), and nine candidate genes, **(D)** Expression of GPR156 and PANX2 in tumor and normal tissues. Based on an analysis of immunohistochemically staining data from the Human Protein Atlas database, the expression of GPR156 and PANX2 in tumor was compared with that in normal tissues.

The risk score was calculated using the abovementioned formula for each patient in TCGA training dataset (RS = (-0.543873) * Exp_CPT2_ + (-0.138679) * Exp_PPARGC1A_ + (0.175724) * Exp_LBX2_ + (0.128094) * Exp_PANX2_ + (0.302522) * Exp_LOC339674_ + (0.11394) * Exp_GPR156_ + (0.233869) * Exp_PPFIA4_ + (0.172185) * Exp_LRP2_ + (0.42619) * Exp_MBL1P_). The optimal cut-off value (cut-off = 4.253) was obtained using the survminer package, which was subsequently used to classify patients in TCGA training dataset into high risk and low risk groups, with grouping details and survival status distribution shown in **Figures 3A, B**; the mortality of the patients who had events was classified in the high-risk group. From the expression levels of the 9-DEGs in the TCGA training dataset (**Figure 3C**), it was observed that PPARG Coactivator 1 Alpha (PPARGC1A) and carnitine palmitoyltransferase 2 (CPT2) had higher expression levels in the low-risk group, whereas the remaining seven genes had higher expression levels in the high-risk group. The same method was also used to calculate the risk scores for TCGA and three GEO validation datasets, which were categorized into high and low-risk groups. The HPA database was used to examine the protein expression of the candidate genes, and IHC staining images of G protein-coupled receptor 156 (GPR156) and pannexin 2 (PANX2) were obtained (**Figure 3D**). GPR156 and PANX2 protein levels were increased in tumor tissues, consistent with their mRNA levels.

### RS signature evaluation

**Figure 4:**
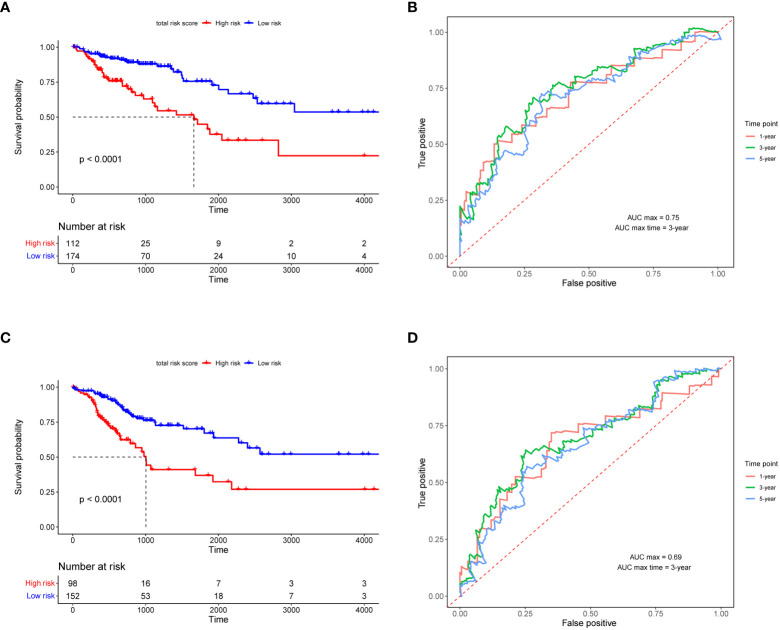
Validation of RS signature. The Kaplan–Meier survival curves **(A, C)** and the ROC curves **(B, D)** of overall survival and disease-free survival for the RS signature in TCGA training dataset. In the KM survival curve, the horizontal coordinate represents the survival time and the vertical coordinate is the survival probability. In the ROC curve, the false positive rate (1-specificity) is the horizontal coordinate and the true positive rate (sensitivity) is the vertical coordinate.

**Figure 5:**
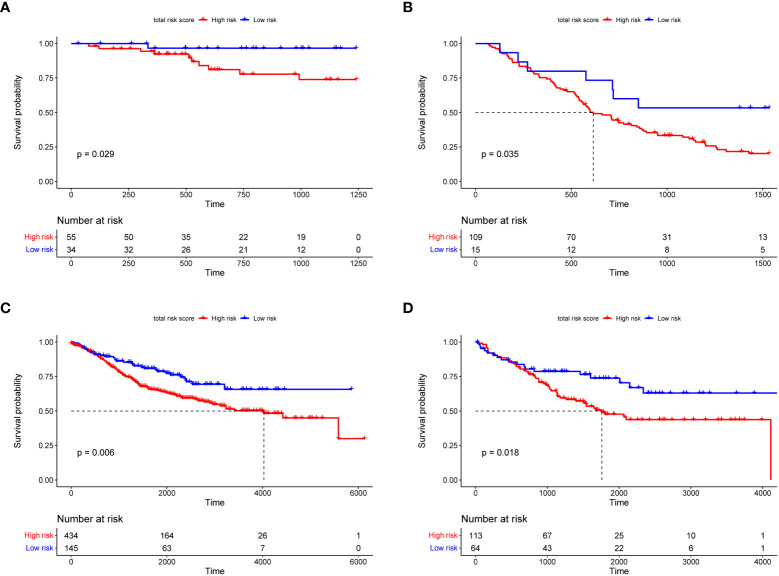
The Kaplan–Meier survival curves of overall survival for the RS signature in TCGA **(A)**, GSE72970 **(B)**, GSE39582 **(C)**, and GSE17536 **(D)** validation datasets. In the KM survival curve, the horizontal coordinate represents the survival time and the vertical coordinate is the survival probability.

**Figure 4** shows the overall survival and disease-free survival of the patients in the training dataset. The overall survival of the low risk group was significantly better than that of the high risk group in the training dataset (*p*< 0.0001, **Figure 4A**), while the 3-year AUC was 0.75 in the ROC curve plot (**Figure 4B**), indicating that the RS signature has excellent predictive accuracy in the TCGA training dataset. In **Figure 4C**, patients in the training dataset classified by the RS signature into the high risk group had a significantly lower probability of being disease-free than those classified into the low risk group (*p*< 0.0001), and with high accuracy (3-year AUC = 0.69, **Figure 4D**). Similarly, the RS signature demonstrated good validation results in the TCGA validation and three GEO independent validation datasets. In all validation datasets, patients in the high-risk group exhibited significantly poorer overall survival compared to those in the low-risk group (*p* = 0.029, *p* = 0.035, *p* = 0.006, and *p* = 0.018, **Figures 5A–D**), which were optimistic predictions of overall patient survival. Furthermore, stage III and IV cancer individuals made up a significant bulk of the GSE72970 validation dataset. As seen in **Figure 5B**, 88% of these patients (n = 109) were categorized into the high-risk group, showing that the RS signature is equally effective at predicting the prognosis of patients with advanced CRC in this study. As shown in **Supplementary Figure 5**, in patients with colon adenocarcinoma (n = 241), the RS signature was a good predictor of the patients’ survival (p = 0.011). While in patients with rectal adenocarcinoma (n = 87), the performance of the RS signature was not as good as it had shown before (p = 0.120), but there was still a trend to differentiate the survival of patients between high and low risk groups.

### Independence of the RS signature for prognostic prediction and the prognostic value of the 9-DEGs

**Figure 6:**
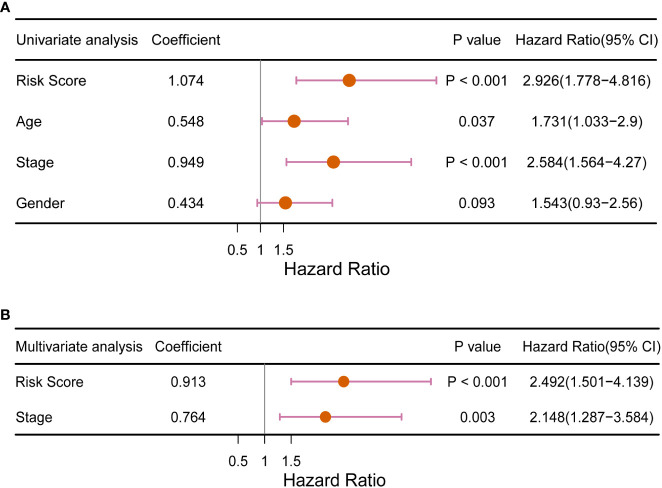
Forest plot of **(A)** univariate and **(B)** multivariate Cox regression analysis of the RS signature compared with traditional clinical factors for its ability to predict the prognosis of CRC patients.

To investigate the ability of RS signature to predict the prognosis of CRC patients compared to other prognostic factors, we performed univariate and multivariate Cox regression analyses with TCGA training dataset, using RS as one of the factors, along with the patient’s age, sex, and cancer pathologic stage. In the univariate Cox regression analysis, we found that both the RS signature and cancer stage were significantly correlated with the prognosis of CRC patients (*p<* 0.001, **Figure 6A**). Subsequently, the RS signature and cancer stage were evaluated for independence (shown in **Figure 6B**), which indicated that the RS signature (*p*< 0.001) is an independent prognostic indicator having a more significant correlation with the prognosis of CRC patients than cancer stage (*p* = 0.003). In DCA (**Supplementary Figure 6**), the AUDC of RS reached 0.023 at 3 years, which was much higher than other clinical indicators (age: 0.002, T: 0.010, N: 0.007, M: 0.010, and TNM stage: 0.006), indicating the high value of RS signature in clinical decision making.

### RS-related DEGs and functional and pathway enrichment analysis

**Figure 7:**
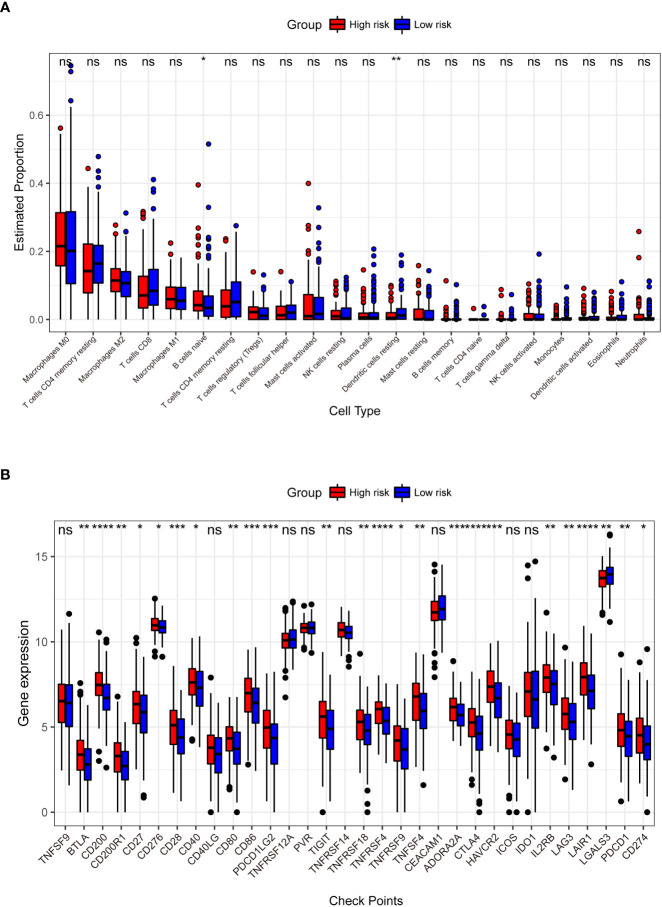
Comparison of the abundance of infiltrating immune cells between high- and low-risk groups **(A)**. The horizontal coordinate is infiltrating immune cells and the vertical coordinate is the estimated proportion. Differences in the expression of 32 immune checkpoints between the high- and low-risk groups **(B)**. The horizontal axis has the 32 immune checkpoints, and the vertical axis indicates the level of expression for each checkpoint. *p< 0.05, **p< 0.01, ***p< 0.001, ****p< 0.0001, ns: not significant.

We identified 853 RS-related DEGs (**Supplementary Table 4**)between the high- and low-risk groups in TCGA training dataset to investigate the differences in gene expression levels between the two groups. As seen in **Supplementary Figures 7A, B**, the majority (n = 798) of the DEGs were down regulated genes, with significant differences in the expression levels between the two groups. There were 18 significant pathways in the KEGG analysis (adjusted *p*< 0.05), with neuroactive ligand-receptor interactions being the most significant (**Supplementary Figure 7C**). In the GO analysis, 489 terms (adjusted *p*< 0.05) were obtained for CC, MF, and BP (100, 54, and 335 terms, respectively). GO analysis results (**Supplementary Figures 7D–F**) revealed that the 9-DEGs were mainly enriched in regulating membrane potential, synaptic membrane, and passive transmembrane transporter activity. In terms of immune infiltration, we discovered a substantial difference between the following two categories of immune cells in the high- and low-risk groups: naïve B cells and resting dendritic cells (**Figure 7A**). There were significant differences in expression between the high- and low-risk groups for 24 of the 32 major immune checkpoints (**Figure 7B**). These include immune checkpoints involved in the immune function of T cells and immune escape mechanisms such as cytotoxic T-lymphocyte associated protein 4 (CTLA4), lymphocyte activation gene 3 (LAG3), T cell immunoreceptor with Ig and ITIM domains (TIGIT), and CD27.

### Drug sensitivity

**Figure 8:**
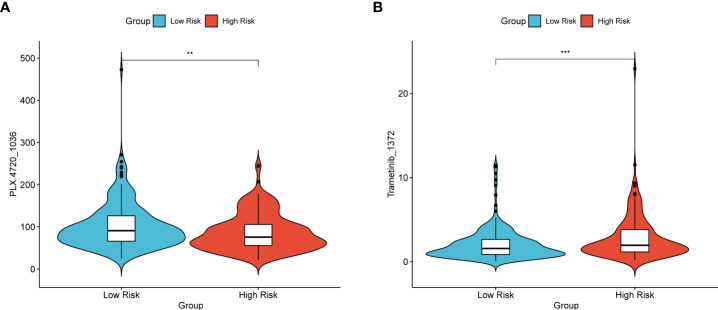
Violin plot of the IC50 of TCGA patients for the two drugs PLX.4720_1036 **(A)** and Trametinib_1372 **(B)**. The vertical axis represents the IC50 values, and the vertical axis is the high- and low-risk groups. *p< 0.05, **p< 0.01, ***p< 0.001, ****p< 0.0001, ns: not significant.

There were two colorectal cancer anticancer drugs with significant differences in IC50 between high and low risk groups in TCGA dataset, PLX.4720_1036 and Trametinib_1372. As shown in **Figure 8**, patients in the low-risk group had a significantly higher IC50 for PLX.4720_1036 (**Figure 8A**) than those in the high-risk group, while Trametinib_ 1372 (**Figure 8B**) showed the opposite result.

## Discussion

Currently, diagnostic methods for CRC are inadequate, resulting in most patients being diagnosed at advanced stages of CRC. Surgical and other treatments are no longer curative, and tumors develop resistance to chemotherapy, worsening the prognosis of CRC patients (30). Colorectal cancer development and advancement are complex biological processes involving several genetic levels and alterations, and several factors influence the rate of disease progression. According to previous research, most cancers are heterogeneous, primarily in pathological type, biological function, and gene alterations, necessitating a tailored therapy for every patient with CRC (31). As a result, classifying patients based on their prognostic risk and implementing distinct individualized treatment strategies for patients with varying risk levels can significantly improve prognosis and prevent tumor recurrence. Therefore, it is crucial to find a reliable method for predicting the prognosis of CRC patients, to accurately assess the effect of treatment promptly and guide further treatment. Developing a risk prediction RS signature is essential for estimating prognosis. This study had two primary goals: the first goal was to construct an RS signature composed of multiple genes in combination to effectively predict the prognosis of CRC patients, because individual biomarkers are not generalizable; and second, was to validate the RS signature using an independent dataset.

Overall, in this study, we screened nine genes that were highly correlated with the prognosis of CRC patients and developed the RS signature, which was validated using multiple independent datasets. The results showed that the RS signature had a strong ability to predict the prognosis of CRC patients. Patients classified as high-risk by the RS signature in all of these datasets had a worse prognosis than those at low-risk. The RS signature can be used as a prognostic indicator for CRC patients.

To establish a credible RS signature, top-down filtering of prognostically significant genes in CRC patients is essential. Using differentially expressed gene analysis, univariate Cox regression analysis, Lasso regression, and multivariate Cox regression analysis, nine characteristic genes that were highly correlated with the prognosis of CRC patients, namely carnitine palmitoyltransferase 2 (*CPT2*), PPARG Coactivator 1 Alpha (*PPARGC1A*), Ladybird Homeobox 2 (*LBX2*), pannexin 2 (*PANX2*), *LOC339674*, G protein-coupled receptor 156 (*GPR156*), PTPRF interacting protein alpha 4 (*PPFIA4*), LDL receptor related protein 2 (*LRP2*), and mannose binding lectin 1, pseudogene (*MBL1P*), were identified for developing the RS signature. Several genes in the RS signature have been shown to be associated with cancers. CPT2 influences tumor development in various cancer, and its low expression correlates with poor prognosis and a worse overall survival rate in patients with CRC (32). PPARGC1A is responsible for regulating several metabolic pathways necessary for tumor cells to respond to microenvironmental changes, thus promoting tumor growth (33–36). LBX2 is upregulated in CRC patients, and its overexpression promotes tumor growth (37). PANX2 is associated with the pathogenesis of prostate cancer and low-grade gliomas in the brain and can affect patient prognosis (38, 39). Overexpression of PPFIA4 promotes tumor progression and reduces the overall survival of patients with CRC (40). These findings suggest that the RS signature developed in this study is strongly associated with tumor progression, and that multiple genes in the RS signature have a large impact on the prognosis of CRC patients.

The RS signature must have a higher predictive power than traditional clinical factors to be of clinical value. The pathological stage of cancer is currently the main criterion for predicting overall survival in patients with CRC (41). At the same time, some studies have confirmed that factors such as age also affect CRC patients (42, 43). In the subsequent overall validation, univariate Cox regression analysis (**Figure 6A**) revealed that only the cancer stage and RS signature were highly correlated with the prognosis of CRC patients (p< 0.01). Multivariate Cox regression analysis (**Figure 6B**) revealed that the RS signature was independent of other clinicopathological factors and had a higher predictive value for the prognosis of CRC patients. The applicability of the RS signature to a large CRC population is another aspect of its clinical value. Three datasets from the GEO database were used to evaluate the general application of this RS signature. In all the three independent validation datasets, the RS signature demonstrated good predictive power for the prognosis of CRC patients; patients classified as high-risk showed significantly lower overall survival compared to low-risk patients in all three datasets (*p* = 0.035, *p* = 0.006, and *p* = 0.018, **Figures 5B–D**). The majority of patients in the GEO GSE72970 dataset had stage III or IV cancer (n = 87), resulting in poor prognosis. After grouping by RS signature, the majority of patients were assigned to the high-risk group (n = 109), with the prognosis of the high-risk group being significantly different from that of the low risk-group (**Figure 5B**). These results demonstrate that the RS signature developed herein, has not just excellent predictive power for the prognosis of CRC patients but is also applicable to a wide range of CRC populations.

To explore the performance of RS signature in different subtypes of patients, we also selected colon and rectal adenocarcinoma patients from TCGA dataset. In patients with rectal adenocarcinoma (n = 87), the performance of the RS signature was not as good as it had shown before (p = 0.120), but there was still a trend to differentiate the survival of patients between high and low risk groups. We considered that the performance of the RS signature in rectal adenocarcinoma patients might be limited by the sample size.

In a recent study, Li and colleges tried to establish a prognostic signature basing on eight genes associated with RNA binding protein using data from TCGA database (44). Since the AUC at 1, 3, and 5 years only reached 0.685, 0.687, and 0.708, respectively, their RS signature did not perform pretty well when predicting the OS of patients. Meanwhile, their signature also did not show better predictive power than traditional clinical indicators. In another study, Liu and colleges construct of an autophagy prognostic model with 13 genes (45). As AUC only reached 0.663 and it had even worse predictive power whether than T, N, M, or TNM stage, their RS signature did not show enough contribution to make convincing predictions on the prognosis of CRC patients. Another prognostic model basing on six metabolism-related genes had a little better performance (46). It had max AUC reached 0.740 at 5 years, however, the performance of their model still did not pull away from the traditional TNM stage. Compared with these previous studies, our RS signature showed a better AUC (max AUC reached 0.750) and possessed greater predictive power than traditional clinical indicators whether in the multivariate Cox regression analysis or DCA. These results revealed its predictive value and application prospects.

KEGG analysis (**Figure 2B**) showed that the cyclic adenosine monophosphate (cAMP) signaling pathway was one of the significant enrichment pathways for the 9-DEGs. All cell proliferation and immunological functions are regulated by this pathway, and its dysfunction or alteration can lead to cancer (47–49). Many of the significant pathways identified in GO analysis were also associated with cancer (**Figures 2C–E**). Disturbances in calcium ion homeostasis lead to apoptosis (50). Lipid raft ion channel complexes increase the migration of colon cancer cells (51). Gated channel activity was found to affect the risk of lung cancer in genome-wide association studies (52). One of the pathways enriched by GSEA, the Wnt signaling pathway, was shown to be a driver of CRC (53), Its misregulation has been reported in many cancers (54).

According to the findings of the previous analysis, there was a substantial difference in overall survival between the high- and low-risk groups into which the patients were classified based on the RS signature. We analyzed the differentially expressed genes in TCGA training dataset between the high- and low-risk groups to investigate the reasons for this difference. The majority of RS-related DEGs were downregulated, as shown in **Supplementary Figures 7A, B**. A previous study (55) examined the transition of normal cells into cancer cells and cancer cell metastasis, and found that the majority of differentially expressed genes (80%) were down regulated. This result supports the prediction ability of the RS signature and suggests that CRC deterioration, which leads to poor patient prognosis, is mostly due to gene turn-off rather than the expression of new genes. The results of the functional and pathway enrichment analysis (**Supplementary Figures 7C–F**) showed that the differentially expressed genes between the high- and low-risk groups were mainly enriched in the calcium signaling pathway, channel activity, synaptic membrane, and regulation of membrane potential. These pathways are associated with apoptosis and cytotoxic immunity (50, 56, 57).

There are multiple types of immune cell infiltration in the tumor microenvironment, and multiple mechanisms suppress immune surveillance. Several suppressive immune receptors/ligands have been identified, and these molecules, which act as gatekeepers for the immune response, are known as immune checkpoints (58, 59). In our analysis, we found significant differences in the expression of 24 immune checkpoints between the high- and low-risk groups (**Figure 7B**). The interaction of CD28, as a T cell surface molecule, with CD80 and CD86 (B7 family) generates a co-stimulatory signal that is critical for T cell clonal expansion and affects its effector functions (60). CTLA4 is an inhibitory receptor that downregulates the initial phase of T cell activation (61) and is expressed primarily in activated and regulatory T cells. CTLA4 is homologous to the T cell surface molecule CD28 and competes with it for binding to the B7 ligand. CTLA4 produces a signal different from that of CD28, and binds to the B7 ligand to inhibit T cells. T cell-mediated immunity is regulated by co-stimulatory and co-inhibitory signals mediated by CD28 and CTLA4 (62). Tumor cell co-stimulatory or co-repressor signaling, involving genes such as *CD28*, *CD80*, *CD86* and *CTLA4*, plays a critical role in regulating cell proliferation, differentiation and cytokine secretion (63). Most B7 family genes are involved in the regulation of tumorigenesis and progression, and abnormal expression of these immune checkpoints fails to effectively induce an anti-tumor immune response, thus evading immune surveillance and playing a critical role in tumor invasion, metastasis, and prognosis (64).. As shown in **Figure 7B**, the expression of these key regulatory genes was significantly higher in patients classified into the high-risk group using the RS signature, further demonstrating the high performance of RS signature in risk-based grouping of CRC patients in this study.

While mining the GDSC database data. The sensitivity to different drugs was found to be different between high and low risk groups. In **Figure 8**, patients in the high-risk group of TCGA had significantly lower resistance to the drug with PLX.4720_1036 (**Figure 8A**), while patients in the low-risk group had significantly lower resistance to Trametinib_ 1372 (**Figure 8B**). This point also illustrates that the RS signature can not only predict the prognosis of CRC patients, but also make judgments about the sensitivity of patients to different drugs, drug resistance, and counsel patients on treatment and diagnosis.

Rather than focusing on a specific type of RNA, we focused on genes that were substantially related to patient prognosis. The findings of this study have significant clinical implications and are predicted to be new indicators for assessing the prognosis of CRC patients. They also provide an important research avenue for experimental investigation of CRC, which is expected to lead to new targets for CRC diagnosis and therapy. Despite our attentive and thorough examination in this study, some issues remain. First, only nine genes were utilized in the creation of the RS signature, resulting in the exclusion of specific important genes, thereby lowering its performance. Second, because the development of CRC is a complicated process involving various components and pathways (3), the use of RNA to build an RS signature to estimate CRC prognosis would produce unsatisfactory results. Third, functional experiments are required to uncover the roles of signature-associated genes and molecular processes in the regulation of CRC progression.

One of the limitations of this study is the cut-off value, which is critical because different cut-offs can lead to patients being assigned to different risk groups. However, there is no accepted gold standard for the cut-off value so far. And the RS signature needs to be validated in a larger sample size cohort and biological experiments can be performed to ensure the clinical use of the RS model.

## Data availability statement

The original contributions presented in the study are included in the article/**Supplementary Material**. Further inquiries can be directed to the corresponding author.

## Author contributions

JC, FG, and YG: conception and design of study. YY, ZM, and YH: acquisition of data. JC, ZM, and YH drafting the manuscript. ZM and YH: analysis and interpretation of data. JC, FG, YG, YY, and JS: revising the manuscript critically for important intellectual content. FG, YY, YG, and JS: approval of the version of the manuscript to be published. All authors contributed to the article and approved the submitted version.

## Acknowledgments

We would like to thank Editage (www.editage.com) for English language editing and all the participants who contributed to this study.

## Conflict of interest

Authors ZM, YH, and JS were employed by ChosenMed Technology Co. Ltd.

The remaining authors declare that the research was conducted in the absence of any commercial or financial relationships that could be construed as a potential conflict of interest.

## Publisher’s note

All claims expressed in this article are solely those of the authors and do not necessarily represent those of their affiliated organizations, or those of the publisher, the editors and the reviewers. Any product that may be evaluated in this article, or claim that may be made by its manufacturer, is not guaranteed or endorsed by the publisher.

## References

[1] Bray F Ferlay J Soerjomataram I Siegel RL Torre LA Jemal A . Global cancer statistics 2018: Globocan estimates of incidence and mortality worldwide for 36 cancers in 185 countries. CA: Cancer J Clin (2018) 68(6):394–424. doi: 10.3322/caac.21492 30207593

[2] Ferlay J Steliarova-Foucher E Lortet-Tieulent J Rosso S Coebergh JW Comber H . Cancer incidence and mortality patterns in Europe: Estimates for 40 countries in 2012. Eur J Cancer (Oxford England: 1990) (2013) 49(6):1374–403. doi: 10.1016/j.ejca.2012.12.027 23485231

[3] Galon J Costes A Sanchez-Cabo F Kirilovsky A Mlecnik B Lagorce-Pagès C . Type, density, and location of immune cells within human colorectal tumors predict clinical outcome. Sci (New York NY) (2006) 313(5795):1960–4. doi: 10.1126/science.1129139 17008531

[4] Goodall GJ Wickramasinghe VO . Rna in cancer. Nat Rev Cancer (2021) 21(1):22–36. doi: 10.1038/s41568-020-00306-0 33082563

[5] Cooper TA Wan L Dreyfuss G . Rna and disease. Cell (2009) 136(4):777–93. doi: 10.1016/j.cell.2009.02.011 PMC2866189 19239895

[6] Wahab R Gopalan V Islam F Mamoori A Lee KT Lu CT . Expression of Gaec1 mrna and protein and its association with clinical and pathological parameters of patients with colorectal adenocarcinoma. Exp Mol Pathol (2018) 104(1):71–5. doi: 10.1016/j.yexmp.2018.01.004 29337242

[7] El-Ashmawy NE Al-Ashmawy GM Hamouda SM . Long non-coding rna Fam83h-As1 as an emerging marker for diagnosis, prognosis and therapeutic targeting of cancer. Cell Biochem Funct (2021) 39(3):350–6. doi: 10.1002/cbf.3601 33159470

[8] Thomas S Prendergast GC . Cancer vaccines: A brief overview. Methods Mol Biol (Clifton NJ) (2016) 1403:755–61. doi: 10.1007/978-1-4939-3387-7_43 27076165

[9] Gao M Kong W Huang Z Xie Z . Identification of key genes related to lung squamous cell carcinoma using bioinformatics analysis. Int J Mol Sci (2020) 21(8):2994. doi: 10.3390/ijms21082994 32340320 PMC7215920

[10] Tang C Ma J Liu X Liu Z . Identification of a prognostic signature of nine metabolism-related genes for hepatocellular carcinoma. PeerJ (2020) 8::e9774. doi: 10.7717/peerj.9774 32953265 PMC7473097

[11] Zhao J Guo C Ma Z Liu H Yang C Li S . Identification of a novel gene expression signature associated with overall survival in patients with lung adenocarcinoma: A comprehensive analysis based on tcga and geo databases. Lung Cancer (Amsterdam Netherlands) (2020) 149:90–6. doi: 10.1016/j.lungcan.2020.09.014 33002836

[12] Ma B Li Y Ren Y . Identification of a 6-lncrna prognostic signature based on microarray re-annotation in gastric cancer. Cancer Med (2020) 9(1):335–49. doi: 10.1002/cam4.2621 PMC6943089 31743579

[13] Lee JH Jung S Park WS Choe EK Kim E Shin R . Prognostic nomogram of hypoxia-related genes predicting overall survival of colorectal cancer-analysis of tcga database. Sci Rep (2019) 9(1):1803. doi: 10.1038/s41598-018-38116-y 30755640 PMC6372658

[14] Wu Z Lu Z Li L Ma M Long F Wu R . Identification and validation of ferroptosis-related lncrna signatures as a novel prognostic model for colon cancer. Front Immunol (2021) 12:783362. doi: 10.3389/fimmu.2021.783362 35154072 PMC8826443

[15] Love MI Huber W Anders S . Moderated estimation of fold change and dispersion for rna-seq data with Deseq2. Genome Biol (2014) 15(12):550. doi: 10.1186/s13059-014-0550-8 25516281 PMC4302049

[16] Wang L Cao C Ma Q Zeng Q Wang H Cheng Z . Rna-seq analyses of multiple meristems of soybean: Novel and alternative transcripts, evolutionary and functional implications. BMC Plant Biol (2014) 14:169. doi: 10.1186/1471-2229-14-169 24939556 PMC4070088

[17] Valero-Mora PM . Ggplot2: Elegant graphics for data analysis. J Stat Software Book Rev (2010) 35(1):1–3. doi: 10.18637/jss.v035.b01

[18] Yu G Wang LG Han Y He QY . Clusterprofiler: An r package for comparing biological themes among gene clusters. Omics: J Integr Biol (2012) 16(5):284–7. doi: 10.1089/omi.2011.0118 PMC3339379 22455463

[19] Carlson M Falcon S Pages H Li NJ . Genome wide annotation for human. Org Hs. Eg. Db (2019) 3(2):3. Rpv.

[20] Wang P Wang Y Hang B Zou X Mao JH . A novel gene expression-based prognostic scoring system to predict survival in gastric cancer. Oncotarget (2016) 7(34):55343–51. doi: 10.18632/oncotarget.10533 PMC5342421 27419373

[21] Friedman JH Hastie T Tibshirani R . Regularization paths for generalized linear models *Via* coordinate descent. J Stat Software (2010) 33(1):1–22. doi: 10.18637/jss.v033.i01 PMC2929880 20808728

[22] Harrell JrFE . Regression modeling strategies. Bios (2017) 330 (2018) :14.

[23] Johnston R Jones K Manley D . Confounding and collinearity in regression analysis: A cautionary tale and an alternative procedure, illustrated by studies of British voting behaviour. Qual quantity (2018) 52(4):1957–76. doi: 10.1007/s11135-017-0584-6 PMC5993839 29937587

[24] Kassambara A Kosinski M Biecek P . Fabian SJRpv. Survminer: Drawing Survival Curves Using ‘Ggplot2’. (2017).

[25] Therneau TM Grambsch PM . *Modeling survival data: Extending the cox model*. New York, NY: Springer. (2013) 2000 :39–77. doi: 10.1007/978-1-4757-3294-8_3

[26] Heagerty PJ Lumley T Pepe MS . Time-dependent roc curves for censored survival data and a diagnostic marker. Biometrics (2000) 56(2):337–44. doi: 10.1111/j.0006-341x.2000.00337.x 10877287

[27] Zhang J . Ggdca: Calculate and plot decision curve. (2022). JZaZ.

[28] Chen B Khodadoust MS Liu CL Newman AM Alizadeh AA . Profiling tumor infiltrating immune cells with cibersort. Methods Mol Biol (Clifton NJ) (2018) 1711:243–59. doi: 10.1007/978-1-4939-7493-1_12 PMC5895181 29344893

[29] Maeser D Gruener RF Huang RS . Oncopredict: An r package for predicting in vivo or cancer patient drug response and biomarkers from cell line screening data. Brief Bioinform (2021) 22(6):bbab260. doi: 10.1093/bib/bbab260 34260682 PMC8574972

[30] Zhang Y Chen Z Li J . The current status of treatment for colorectal cancer in China: A systematic review. Medicine (2017) 96(40):e8242. doi: 10.1097/md.0000000000008242 28984783 PMC5738019

[31] Dekker E Tanis PJ Vleugels JLA Kasi PM Wallace MB . Colorectal cancer. Lancet (London England) (2019) 394(10207):1467–80. doi: 10.1016/s0140-6736(19)32319-0 31631858

[32] Lin AP Huang TW Tam KW . Treatment of Male breast cancer: Meta-analysis of real-world evidence. Br J Surg (2021) 108(9):1034–42. doi: 10.1093/bjs/znab279 34476472

[33] Shlomi T Benyamini T Gottlieb E Sharan R Ruppin E . Genome-scale metabolic modeling elucidates the role of proliferative adaptation in causing the warburg effect. PloS Comput Biol (2011) 7(3):e1002018. doi: 10.1371/journal.pcbi.1002018 21423717 PMC3053319

[34] Deberardinis RJ Sayed N Ditsworth D Thompson CB . Brick by brick: Metabolism and tumor cell growth. Curr Opin Genet Dev (2008) 18(1):54–61. doi: 10.1016/j.gde.2008.02.003 18387799 PMC2476215

[35] Bhalla K Hwang BJ Dewi RE Ou L Twaddel W Fang HB . Pgc1α promotes tumor growth by inducing gene expression programs supporting lipogenesis. Cancer Res (2011) 71(21):6888–98. doi: 10.1158/0008-5472.Can-11-1011 PMC3282487 21914785

[36] Alix-Panabières C Cayrefourcq L Mazard T Maudelonde T Assenat E Assou S . Molecular portrait of metastasis-competent circulating tumor cells in colon cancer reveals the crucial role of genes regulating energy metabolism and DNA repair. Clin Chem (2017) 63(3):700–13. doi: 10.1373/clinchem.2016.263582 28007957

[37] Huang X Yang Y Yang C Li H Cheng H Zheng Y . Overexpression of Lbx2 associated with tumor progression and poor prognosis in colorectal cancer. Oncol Lett (2020) 19(6):3751–60. doi: 10.3892/ol.2020.11489 PMC7202318 32382328

[38] Liao D Yang G Yang Y Tang X Huang H Shao J . Identification of pannexin 2 as a novel marker correlating with ferroptosis and malignant phenotypes of prostate cancer cells. OncoTargets Ther (2020) 13:4411–21. doi: 10.2147/ott.S249752 PMC7245471 32547072

[39] Xu X Hao Y Xiong S He Z . Panx2 and brain lower grade glioma genesis: A bioinformatic analysis. Sci Prog (2021) 104(2):368504211011836. doi: 10.1177/00368504211011836 33913372 PMC10305810

[40] Huang J Yang M Liu Z Li X Wang J Fu N . Ppfia4 promotes colon cancer cell proliferation and migration by enhancing tumor glycolysis. Front Oncol (2021) 11:653200. doi: 10.3389/fonc.2021.653200 34094943 PMC8173052

[41] Bosch LJ Carvalho B Fijneman RJ Jimenez CR Pinedo HM van Engeland M . Molecular tests for colorectal cancer screening. Clin colorectal Cancer (2011) 10(1):8–23. doi: 10.3816/CCC.2011.n.002 21609931

[42] Wang T Maden SK Luebeck GE Li CI Newcomb PA Ulrich CM . Dysfunctional epigenetic aging of the normal colon and colorectal cancer risk. Clin Epigenet (2020) 12(1):5. doi: 10.1186/s13148-019-0801-3 PMC6942339 31900199

[43] Bailey CE Hu CY You YN Bednarski BK Rodriguez-Bigas MA Skibber JM . Increasing disparities in the age-related incidences of colon and rectal cancers in the united states, 1975-2010. JAMA Surg (2015) 150(1):17–22. doi: 10.1001/jamasurg.2014.1756 25372703 PMC4666003

[44] Li T Hui W Halike H Gao F . Rna binding protein-based model for prognostic prediction of colorectal cancer. Technol Cancer Res Treat (2021) 20:15330338211019504. doi: 10.1177/15330338211019504 34080453 PMC8182183

[45] Liu TT Liu SM . Prediction of prognostic biomarkers and construction of an autophagy prognostic model for colorectal cancer using bioinformatics. Technol Cancer Res Treat (2020) 19:1533033820984177. doi: 10.1177/1533033820984177 33357130 PMC7780303

[46] Sun YL Zhang Y Guo YC Yang ZH Xu YC . A prognostic model based on six metabolism-related genes in colorectal cancer. BioMed Res Int (2020) 2020:5974350. doi: 10.1155/2020/5974350 32953885 PMC7482003

[47] Lefkimmiatis K Zaccolo M . Camp signaling in subcellular compartments. Pharmacol Ther (2014) 143(3):295–304. doi: 10.1016/j.pharmthera.2014.03.008 24704321 PMC4117810

[48] Fernandes HB Riordan S Nomura T Remmers CL Kraniotis S Marshall JJ . Epac2 mediates camp-dependent potentiation of neurotransmission in the hippocampus. J neuroscience: Off J Soc Neurosci (2015) 35(16):6544–53. doi: 10.1523/jneurosci.0314-14.2015 PMC4405561 25904804

[49] Sirotkin AV Ben OA Tandlmajerová A Lauková M Vaší Ek D Laurin Ik J . Camp response element-binding protein 1 controls porcine ovarian cell proliferation, apoptosis, and fsh and insulin-like growth factor 1 response. Reproduction fertility Dev (2018) 30(8):1145–53. doi: 10.1071/rd17508 29448973

[50] Park H Lim W You S Song G . Oxibendazole induces apoptotic cell death in proliferating porcine trophectoderm and uterine luminal epithelial cells *Via* mitochondria-mediated calcium disruption and breakdown of mitochondrial membrane potential. Comp Biochem Physiol Toxicol pharmacology: CBP (2019) 220:9–19. doi: 10.1016/j.cbpc.2019.02.014 30822534

[51] Guéguinou M Harnois T Crottes D Uguen A Deliot N Gambade A . Sk3/Trpc1/Orai1 complex regulates soce-dependent colon cancer cell migration: A novel opportunity to modulate anti-egfr mab action by the alkyl-lipid ohmline. Oncotarget (2016) 7(24):36168–84. doi: 10.18632/oncotarget.8786 PMC5094991 27102434

[52] Ji X Bossé Y Landi MT Gui J Xiao X Qian D . Identification of susceptibility pathways for the role of chromosome 15q25.1 in modifying lung cancer risk. Nat Commun (2018) 9(1):3221. doi: 10.1038/s41467-018-05074-y 30104567 PMC6089967

[53] Schatoff EM Leach BI Dow LE . Wnt signaling and colorectal cancer. Curr Colorectal Cancer Rep (2017) 13(2):101–10. doi: 10.1007/s11888-017-0354-9 PMC5391049 28413363

[54] Caspi M Wittenstein A Kazelnik M Shor-Nareznoy Y Rosin-Arbesfeld R . Therapeutic targeting of the oncogenic wnt signaling pathway for treating colorectal cancer and other colonic disorders. Adv Drug Delivery Rev (2021) 169:118–36. doi: 10.1016/j.addr.2020.12.010 33346022

[55] Danielsson F Skogs M Huss M Rexhepaj E O’Hurley G Klevebring D . Majority of differentially expressed genes are down-regulated during malignant transformation in a four-stage model. Proc Natl Acad Sci United States America (2013) 110(17):6853–8. doi: 10.1073/pnas.1216436110 PMC3637701 23569271

[56] Kabanova A Zurli V Baldari CT . Signals controlling lytic granule polarization at the cytotoxic immune synapse. Front Immunol (2018) 9:307. doi: 10.3389/fimmu.2018.00307 29515593 PMC5826174

[57] Pei JV Heng S De Ieso ML Sylvia G Kourghi M Nourmohammadi S . Development of a photoswitchable lithium-sensitive probe to analyze nonselective cation channel activity in migrating cancer cells. Mol Pharmacol (2019) 95(5):573–83. doi: 10.1124/mol.118.115428 30858164

[58] Wang W Kryczek I Dostál L Lin H Tan L Zhao L . Effector T cells abrogate stroma-mediated chemoresistance in ovarian cancer. Cell (2016) 165(5):1092–105. doi: 10.1016/j.cell.2016.04.009 PMC4874853 27133165

[59] Gandhi L Rodríguez-Abreu D Gadgeel S Esteban E Felip E De Angelis F . Pembrolizumab plus chemotherapy in metastatic non-Small-Cell lung cancer. New Engl J Med (2018) 378(22):2078–92. doi: 10.1056/NEJMoa1801005 29658856

[60] Nagai S Azuma M . The Cd28-B7 family of Co-signaling molecules. Adv Exp Med Biol (2019) 1189:25–51. doi: 10.1007/978-981-32-9717-3_2 31758530

[61] Son CH Bae JH Shin DY Lee HR Choi YJ Jo WS . Ctla-4 blockade enhances antitumor immunity of intratumoral injection of immature dendritic cells into irradiated tumor in a mouse colon cancer model. J immunotherapy (Hagerstown Md: 1997) (2014) 37(1):1–7. doi: 10.1097/cji.0000000000000007 24316550

[62] Christian M Cermak T Doyle EL Schmidt C Zhang F Hummel A . Targeting DNA double-strand breaks with tal effector nucleases. Genetics (2010) 186(2):757–61. doi: 10.1534/genetics.110.120717 PMC2942870 20660643

[63] Karimi P Islami F Anandasabapathy S Freedman ND Kamangar F . Gastric cancer: Descriptive epidemiology, risk factors, screening, and prevention. Cancer epidemiology Biomarkers prevention: Publ Am Assoc Cancer Research cosponsored by Am Soc Prev Oncol (2014) 23(5):700–13. doi: 10.1158/1055-9965.Epi-13-1057 PMC4019373 24618998

[64] Chen R Ganesan A Okoye I Arutyunova E Elahi S Lemieux MJ . Targeting B7-1 in immunotherapy. Medicinal Res Rev (2020) 40(2):654–82. doi: 10.1002/med.21632 31448437

