# Supplementary material for: Development and validation of a prognostic 9-gene signature for colorectal cancer
Source: Front Oncol. 2022 Nov 17;12:1009698. doi: 10.3389/fonc.2022.1009698 (PMC9714635; doi:10.3389/fonc.2022.1009698)
Supplement: Supplementary file 1 [file DataSheet_1.docx]

***Supplementary Material***

**1. Supplementary Figures**


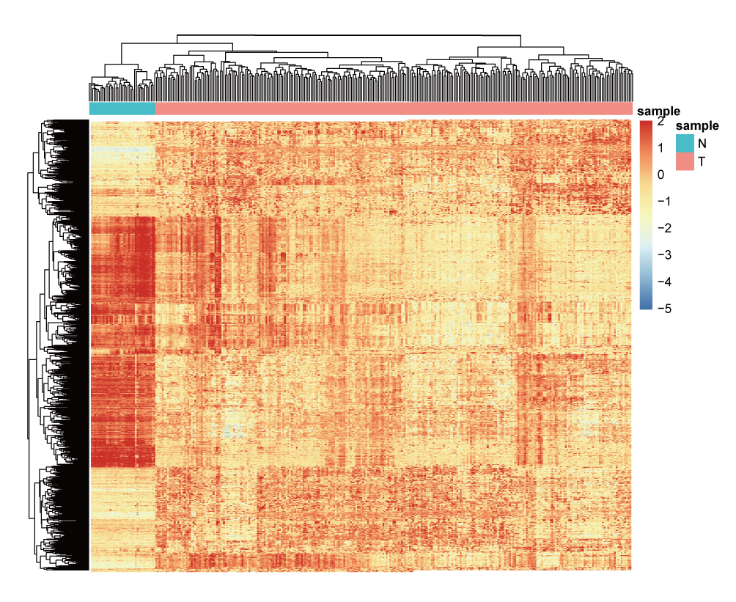


**Supplementary Figure 1.** Heatmap of top 100 DEGs between tumor (T) samples and normal (N) tissue samples in the TCGA training dataset. The horizontal coordinate is the sample and the vertical coordinate is the differentially expressed gene, from red to blue represents a gradual decrease in gene expression.


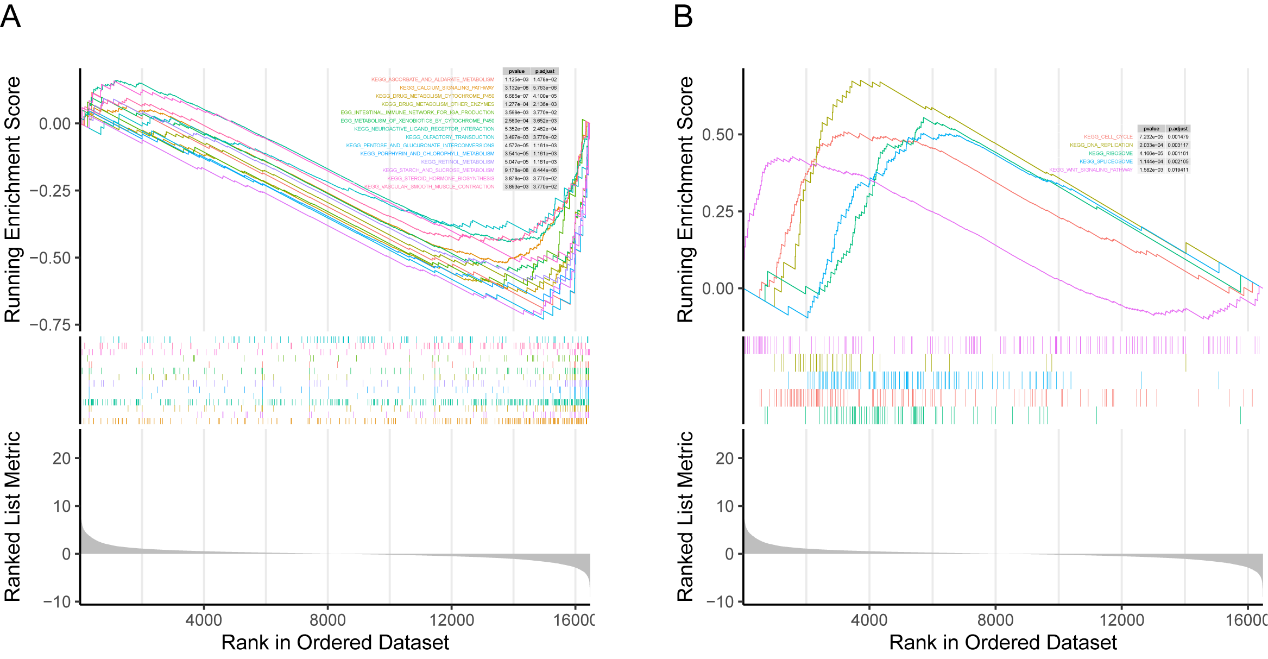


**Supplementary Figure 2.** The GSEA results of (A) low-expressed genes and (B) high-expressed genes.


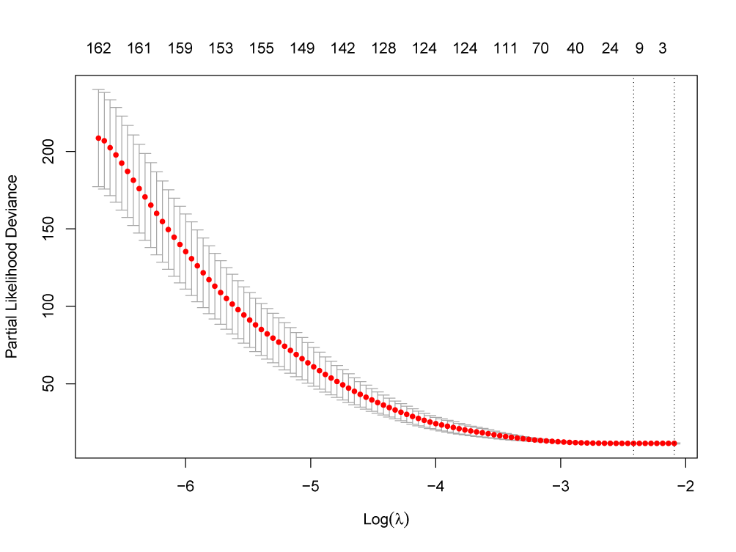


**Supplementary Figure 3.** Parameter selection in the LASSO regression signature. The two dashed lines are the value of λ at the minimum of the mean square error and the value of λ at one standard error away from the minimum of the mean square error


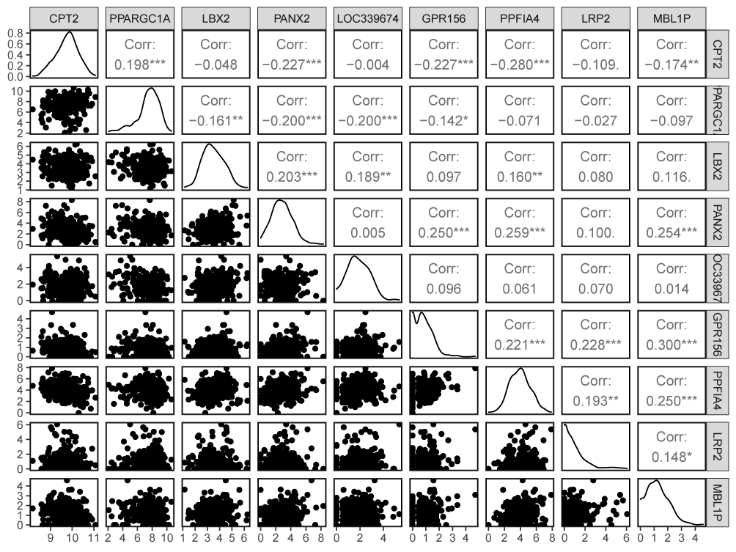


**Supplementary Figure 4.** 9-DEGs correlation matrix.


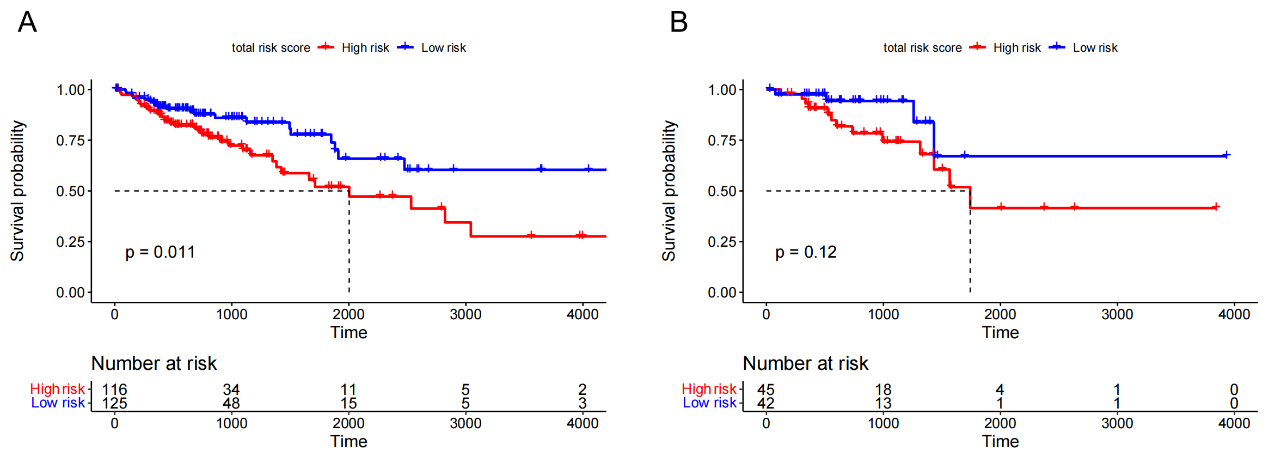


**Supplementary Figure 5.** The Kaplan–Meier survival curves of overall survival for the RS signature in colon adenocarcinoma patients (A) and rectal adenocarcinoma patients (B) from TCGA. In the KM survival curve, the horizontal coordinate represents the survival time and the vertical coordinate is the survival probability.


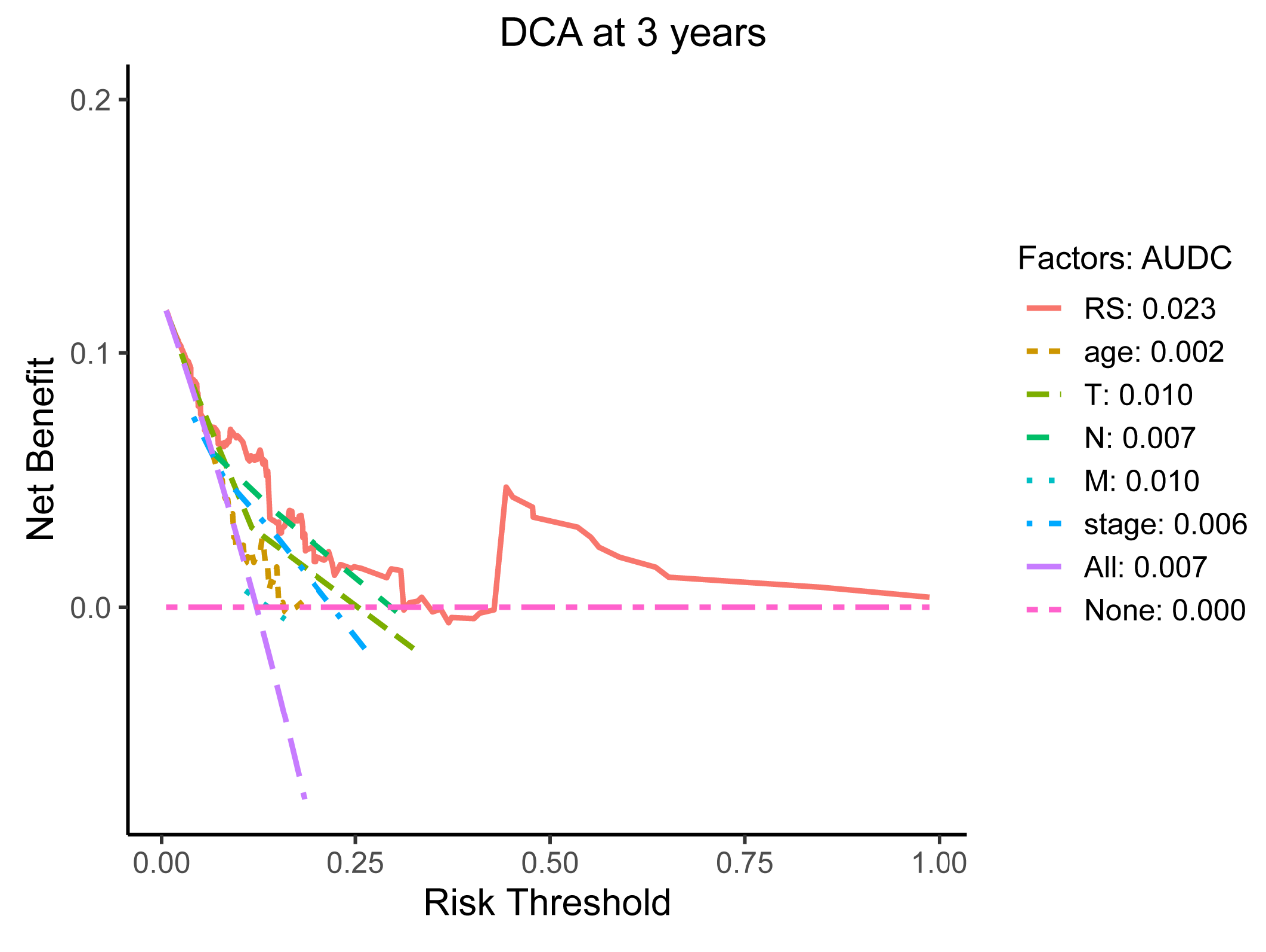


**Supplementary Figure 6.** The DCA curves of overall survival and other clinical indicators (including age, sex, tumor (T), node (N), metastasis (M), and TNM stage) at 3 years. The area under decision curve (AUDC) represents the area enclosed between the DCA curve of each indicator and None and All, which reflects the total net benefit of RS and other indicators.


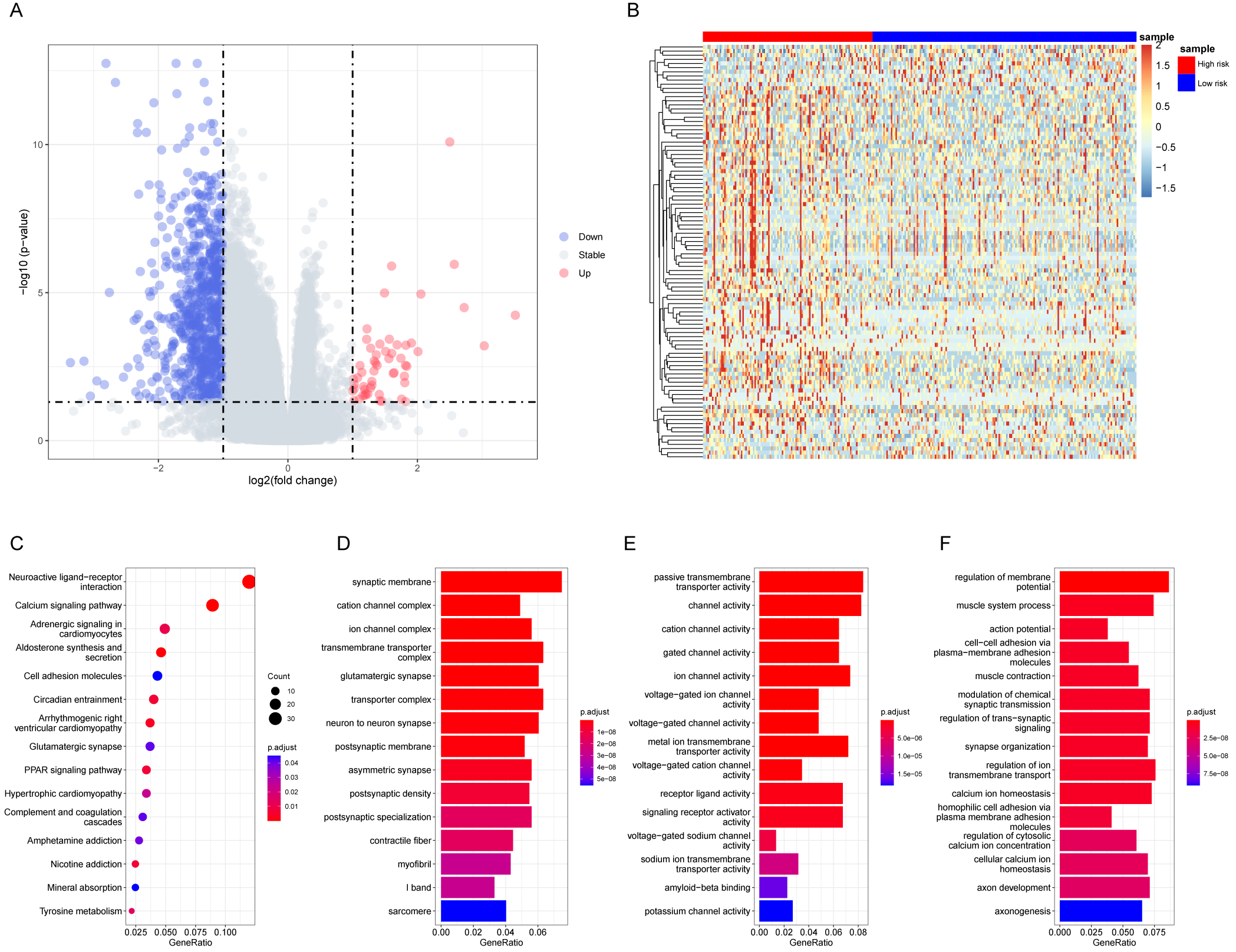


**Supplementary Figure 7.** (A) Volcano plot of risk score-related DEGs in the TCGA training dataset., the horizontal coordinate is log2 (fold change), with the distribution of genes with large differences at either end of the horizontal coordinate. The vertical coordinate is -log10 (p-value), with larger p-values indicating more significant differences. (B) heatmap of risk score-related DEGs, the horizontal coordinate is the sample and the vertical coordinate is the differentially expressed gene, from red to blue represents a gradual decrease in gene expression. (C) KEGG analysis, and histograms of (D) cellular component, (E) molecular function, and (F) biological process in GO analysis of risk score-related DEGs in the TCGA training dataset. The horizontal coordinate is generated, representing the ratio of the pathway’s or term’s genes to the total genes. The vertical coordinate is the name of the pathways or terms enriched to. The color represents the *p*-value, the redder the color the larger the *p*-value.

**2. Supplementary Tables**

**Supplementary Table 1.** 32 key immune checkpoints.

**Supplementary Table 2.** Differential expression of genes between tumor samples and normal samples in the TCGA training dataset.

**Supplementary Table 3.** Comparison of clinical indicators between training dataset and validation dataset in TCGA.

**Supplementary Table 4.** Differential expression of genes between high and low risk groups in the TCGA training dataset.
